# Image-Guided Embolization Coil Placement for Identification of an Endophytic, Isoechoic Renal Mass During Robotic Partial Nephrectomy

**DOI:** 10.1089/cren.2015.0022

**Published:** 2015-11-01

**Authors:** James Jeffery Reeves, Andrew Forauer, John D. Seigne, Elias S. Hyams

**Affiliations:** ^1^ Geisel School of Medicine, Dartmouth College, Hanover, New Hampshire.; ^2^ Division of Vascular and Interventional Radiology, Department of Radiology, Dartmouth-Hitchcock Medical Center, Lebanon, New Hampshire.; ^3^ Section of Urology, Department of Surgery, Dartmouth-Hitchcock Medical Center, Lebanon, New Hampshire.

## Abstract

***Background:*** Intraoperative ultrasonography has proven to be a useful tool for tumor identification during robot-assisted laparoscopic partial nephrectomy (RALPN). However, its utility is limited in renal tumors that are completely endophytic and isoechoic in nature. We present a novel approach to intraoperative tumor identification using preoperative percutaneous intratumoral embolization coil placement that may be utilized in the management of such cases.

***Case Presentation:*** A 42-year-old Caucasian male was referred with an incidentally discovered right renal mass that was posterior and completely endophytic. He desired a RALPN; however, preoperative renal ultrasound demonstrated an isoechoic lesion. Thus, the patient underwent preoperative image-guided placement of an embolization coil within the tumor. This facilitated identification of the tumor intraoperatively using intracorporeal ultrasound centered on the coil and enabled resection with negative margins.

***Conclusion:*** Utilizing a novel approach analogous to preoperative localization of other solid malignancies, such as breast cancer, we were able to effectively identify and resect an isoechoic renal mass during RALPN.

## Introduction

The use of intraoperative ultrasonography has proven to be effective in tumor identification during robot-assisted laparoscopic partial nephrectomy (RALPN).^1^ However, in a subset of renal tumors that are completely endophytic and isoechoic in nature, the utility of intracorporeal ultrasound is limited. Herein we present a novel approach in which preoperative image-guided placement of an embolization coil within an isoechoic tumor was utilized to facilitate identification and subsequent resection with negative margins.

## Case Presentation

**FIG. 1.**
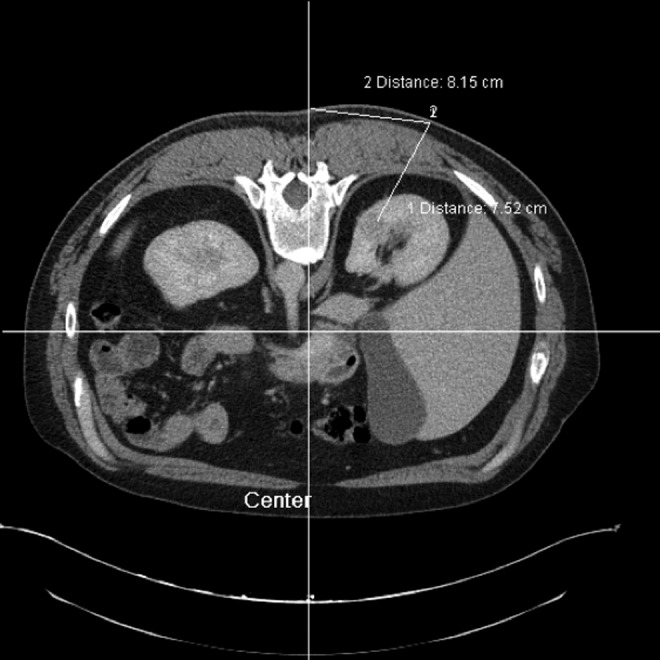
Endophytic renal mass on posterior right kidney.

A 42-year-old male patient was referred with an incidentally discovered right renal mass. He originally presented with acute epigastric pain and was found to have acute pancreatitis on CT. The scan also demonstrated a 2.6 cm heterogeneously enhancing right renal mass concerning for neoplasm. The mass was entirely endophytic, located along the medial aspect of the upper pole (Fig. 1). After resolution of pancreatitis, a percutaneous renal biopsy was performed that demonstrated clear-cell renal cell carcinoma (ccRCC), Fuhrman grade 2. Treatment options were reviewed with the patient, including partial nephrectomy, both open and minimally invasive, ablative therapies, including radiofrequency ablation and cryoablation, and radical nephrectomy. Based on a desire for a more definitive oncologic procedure and a more rapid recovery, he elected for an RALPN with the possibility of open conversion. A renal ultrasound was performed to confirm that the tumor was identifiable on sonography, as the tumor would require intraoperative identification with ultrasound based on its endophytic nature; however, the tumor was found to be isoechoic. He then elected to proceed with a novel procedure for intraoperative tumor identification, that is, percutaneous placement of a localizing vascular embolization coil by interventional radiology (IR) preoperatively, which might facilitate RALPN.

**FIG. 2.**
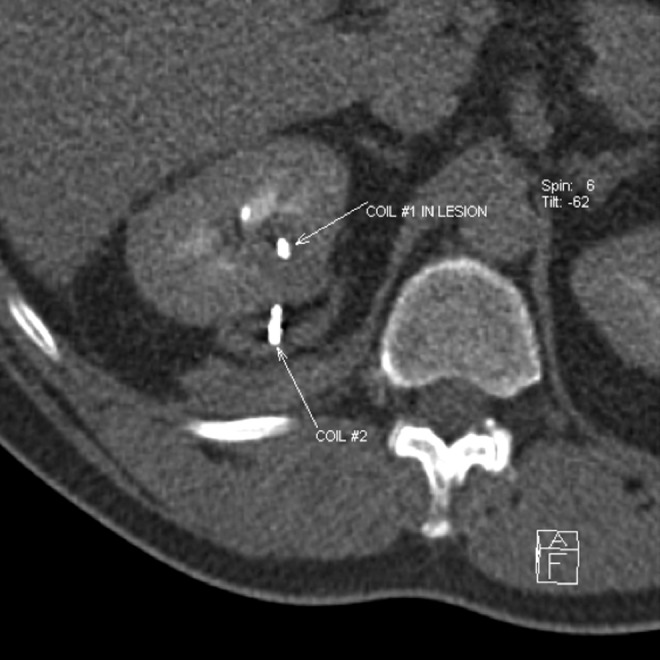
Percutaneously placed metal coils within and abutting right renal mass.

**FIG. 3.**
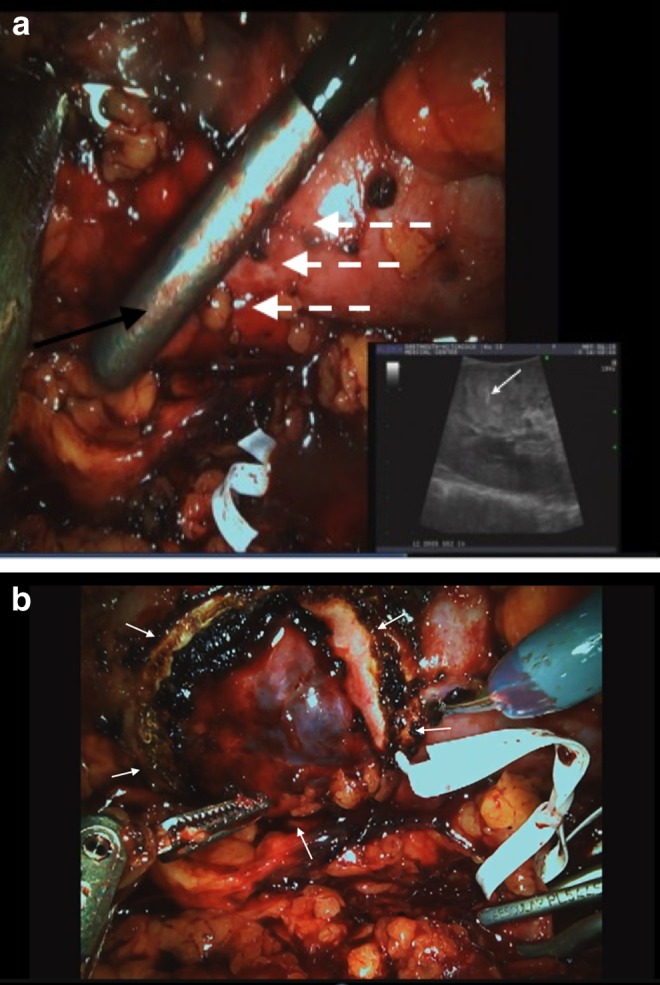
**(a)** Intracorporeal laparoscopic ultrasound demonstrating metal coil within indistinct, endophytic, isoechoic renal mass. *Dashed arrows*: area of suspected endophytic mass along medial aspect of the upper pole; *Black arrow*: ultrasound probe; *White arrow*: hyperechoic changes and artifact of metallic coil on ultrasound. **(b)** Capsular marking of anticipated tumor excision borders, marked with cautery using laparoscopic ultrasound as a guide. *Arrows* outline marking.

The morning of surgery, he underwent coil placement by IR; a 2 × 5 mm platinum embolization coil was percutaneously deployed under CT guidance into the center of the tumor through a 22-gauge ×20-cm Chiba needle (Fig. 2). A second coil was placed at the capsule of the tumor to further guide in identification. The patient was then taken for retroperitoneal RALPN. The retroperitoneal space was insufflated and ports were placed in a standard manner. The renal artery and then vein were identified. The kidney was defatted and the capsule was exposed. Consistent with preoperative imaging, there was no exophytic portion of the tumor. The capsular coil could not be seen and was presumed to have been dislodged during dissection of perirenal fat. However, when laparoscopic ultrasound was performed, the intratumoral coil could be visualized by the metallic artifact on the ultrasonic image (Fig. 3a). Intended lines of resection were marked on the capsule (Fig. 3b). Bulldog clamps were placed on the renal artery and renal vein, the kidney blanched, and the tumor was resected with grossly negative margins. The collecting system was entered. The defect was over sewn in standard manner, the clamps were removed, and hemostasis was achieved. The procedure was completed and the patient had no complications. Pathology revealed a 2.5 cm ccRCC, Fuhrman grade 2 with negative margins.

## Discussion

This case presented unique management challenges for partial nephrectomy in a patient with a central, endophytic, and isoechoic renal mass who strongly desired a robot-assisted laparoscopic approach. The central and deep position made ablation more problematic based on risk of injury to central structures,^2^ and he desired extirpative surgery for a more definitive oncologic result. While he accepted the possibility of requiring open surgery, he was young and active and desired a minimally invasive approach if possible.

The particular challenge of this case was the tumor's isoechoic appearance and totally endophytic position. As mentioned earlier, the utility of intraoperative ultrasonography is limited with isoechoic tumors and renders these cases more challenging. We undertook a novel approach of preoperative CT-guided coil placement to enable intraoperative identification of the tumor. The platinum coil exhibited a characteristic hyperechoic appearance on ultrasound, aiding in identification of the otherwise isoechoic tumor. This allowed for a confident robotic resection with negative surgical margins. Our approach is analogous to localization techniques commonly used in surgical therapy of nonpalpable breast cancer,^3^ although an analogous technique has not been reported for resection of renal masses based on a detailed literature search. We believe this approach may be useful for similar masses in the future, although challenges include the need for an experienced IR team that can place a coil with a three-dimensional understanding of the tumor, as well as the need for two anesthesia settings, although the latter may not be required depending on hospital logistics. Ultimately, it was the intratumoral coil that enabled detection and resection of the tumor; the capsular coil likely dislodged during dissection within the perirenal fat. Given the limited working space of the retroperitoneum and the need for retroperitoneal dissection, a larger coil or wire was not practical; however, further investigation of capsular identifiers of endophytic tumors would be potentially useful.

## Conclusion

We present a novel approach in the management of a patient with an endophytic and isoechoic renal tumor who desired RALPN. The utilization of preoperative image-guided embolization coil placement within the tumor enabled identification and confident surgical resection of this patient's mass. This approach may be useful for similar cases in the future.

## Abbreviations Used

ccRCC: clear-cell renal cell carcinoma
CT: computed tomography
IR: interventional radiology
RALPN: robot-assisted laparoscopic partial nephrectomy
